# Impact of Frailty on Short-Term Outcomes After Laparoscopic and Open Hepatectomy

**DOI:** 10.1007/s00268-022-06648-0

**Published:** 2022-07-09

**Authors:** D. Osei-Bordom, L. Hall, J. Hodson, K. Joshi, L. Austen, D. Bartlett, J. Isaac, D. F. Mirza, R. Marudanayagam, K. Roberts, B. V. Dasari, N. Chatzizacharias, R. P. Sutcliffe

**Affiliations:** 1 Department of General Surgery, Queen Elizabeth Hospital Birmingham, University Hospitals Birmingham NHS Foundation Trust, Birmingham, B15 2TH UK; 2 Institute of Immunology and Immunotherapy, College of Medical and Dental Sciences, University of Birmingham, Birmingham, B15 2TH UK; 3 College of Medical and Dental Sciences, University of Birmingham, Birmingham, B15 2TH UK; 4 Department of Medical Statistics, Institute of Translational Medicine, University Hospitals Birmingham NHS Foundation Trust, Birmingham, B15 2TH UK; 5 The Liver Unit, Nuffield House, Queen Elizabeth Hospital Birmingham, University Hospitals Birmingham NHS Foundation Trust, Birmingham, B15 2TH UK; 6 Department of Anaesthesia, Birmingham Heartlands Hospital, University Hospitals Birmingham NHS Foundation Trust, Birmingham, B9 5SS UK

## Abstract

**Background:**

Although laparoscopic hepatectomy (LH) is associated with improved short-term outcomes compared to open hepatectomy (OH), it is unknown whether frail patients also benefit from LH. The aim of this study was to evaluate the impact of frailty on post-operative outcomes after LH and OH.

**Patients and methods:**

Consecutive patients who underwent LH and OH between January 2011 and December 2018 were identified from a prospective database. Frailty was assessed using the modified Frailty Index (mFI), with patients scoring mFI ≥ 1 deemed to be frail.

**Results:**

Of 1826 patients, 34.7% (*N* = 634) were frail and 18.6% (*N* = 340) were elderly (≥ 75 years). Frail patients had significantly higher 90-day mortality (6.6% vs. 2.9%, *p* < 0.001) and post-operative complications (36.3% vs. 26.1%, *p* < 0.001) than those who were not frail, effects that were independent of patient age on multivariate analysis. For those undergoing minor resections, the benefits of LH vs. OH were similar for frail and non-frail patients. Length of hospital stay was 53% longer in OH (vs. LH) in frail patients, compared to 58% longer in the subgroup of non-frail patients.

**Conclusions:**

Frailty is independently associated with inferior post-operative outcomes in patients undergoing hepatectomy. However, the benefits of laparoscopic (compared to open) hepatectomy are similar for frail and non-frail patients. Frailty should not be a contraindication to laparoscopic minor hepatectomy in carefully selected patients.

## Introduction

Hepatectomy is a potentially curative treatment for patients with primary and secondary hepatic malignancies, and a laparoscopic approach, when feasible, is associated with less morbidity, lower mortality and faster recovery than open surgery [1, 2]. In an increasingly ageing population [3], there is a growing number of frail and elderly patients with comorbidity who are being considered for major surgery, including hepatectomy [4]. Although the risks of surgery are known to increase with advancing age, the short-term advantages of laparoscopic hepatectomy (LH) compared to open hepatectomy (OH) appear to be retained, at least in selected patients undergoing minor hepatectomy [5, 6]. However, the benefits of LH appear to diminish with increasing age amongst elderly cohorts [7], and it is not known whether similar benefits of LH are observed in frail patients.

Frailty is a condition that is characterised by reduced physiological reserve and is associated with an increased risk of post-operative complications, prolonged hospitalization, increased readmission rates and loss of independence following general surgical procedures [8]. Frailty is also common in patients undergoing hepatectomy, with a reported incidence of 14–29% in recently published studies [9, 10], although there is currently limited data to support an association with worse post-operative outcomes [10]. Available data on the impact of frailty in minimally invasive surgery is also limited and conflicting. In two recent studies of the ACS-NSQIP database, Kothari et al. demonstrated that the benefits of laparoscopic colectomy over open colectomy were preserved in frail patients [11], whilst Lo et al. reported possible worse outcomes after robotic compared to open colectomy [12]. The effects of frailty on the short-term outcomes of LH are unknown, and it is unclear whether frail patients should preferentially undergo LH or OH.

Various clinical scores have been developed and validated to measure frailty in surgical patients, and typically include an assessment of functional status combined with the presence and severity of comorbidity, such as diabetes mellitus, hypertension and cardiovascular disease [13]. The modified Frailty Index (mFI) [14] consists of 11 variables that were adapted from the original 70-item Frailty Index [15] and has been shown to be significantly associated with poor surgical outcomes, including after hepatectomy [8].

The primary aim of this study was to evaluate the effects of age and frailty on short-term post-operative outcomes after hepatectomy. The secondary aim was to evaluate the relationship between age, frailty and post-operative outcomes in patients undergoing laparoscopic and open hepatectomy.

## Methods

Consecutive patients who underwent laparoscopic or open hepatectomy at a single centre between 2011 and 2018 were identified from a prospectively maintained database. Repeat hepatectomy (*N* = 152) and emergency hepatectomy for trauma (*N* = 4) patients were excluded. The type of hepatectomy was defined as minor, major or extra major according to the Tokyo 2020 terminology of liver anatomy and resections which is an update of the Brisbane 2000 system [16]. Frailty was defined as a modified Frailty Index (mFI) ≥ 1 and was calculated for all patients. Patients aged 75 years and over were considered elderly, and the effects of both frailty and age on hepatectomy outcomes were compared. Data were collected regarding comorbidity, indications for surgery, post-operative complications, length of hospital (LOS) and 90-day mortality. Post-operative complications were graded according to the Clavien–Dindo classification [17].

### Statistical analysis

Continuous variables were reported as median (interquartile range; IQR), and associations with age and mFI were assessed using Mann–Whitney U tests. Ordinal variables were analysed using the same approach, whilst nominal variables were assessed using Fisher’s exact tests. To assess the interplay between age and frailty with respect to dichotomous patient outcomes, binary logistic regression models were produced with age, frailty and the age*frailty interaction as independent variables. These models were then evaluated to produce odds ratios for frail vs. non-frail patients within each age subgroup, with the *p*-value of the interaction term representing the comparison between these two odds ratios. Length of stay was then analysed using a similar approach, but using an ANOVA model. Lengths of stay were log_10_-transformed, prior to this analysis, in order to normalise the distribution; hence were summarised using geometric means, with differences between groups reported as percentages. Multivariable models were then produced, to assess whether age and frailty were independent predictors of the primary outcomes. This used binary logistic regression models for 90-day mortality and complication rates, with a general linear model used for length of stay, which was log_10_-transformed for analysis. Age and frailty were entered into the models as continuous covariates, and a backwards stepwise approach was used to select other potentially confounding factors for inclusion in the models. Analyses were then performed to compare the effect of operative approach (open vs. laparoscopic) between subgroups of age and frailty, which used a similar approach to that previously described. All analyses were performed using IBM SPSS 24 (IBM Corp. Armonk, NY), with *p* < 0.05 deemed to be indicative of statistical significance throughout.

## Results

### Cohort characteristics

Data were available for a total of *N* = 1826 patients (56.6% male), with a median age of 65.3 years (IQR: 56.0–73.1). The most common indication for surgery was colorectal liver metastases (58.8%), with the majority of patients undergoing open surgery (86.0%). The extent of resection was minor in 53.1% of cases, with 33.3% major and 13.6% extra-major resections. Major (Clavien–Dindo grade III-V) complications developed in 10.7% of patients, and patients surviving to discharge had a median length of stay of 6 days (IQR: 5–8). In-hospital, 30-day and 90-day mortality rates were 2.2% (*N* = 40), 1.9% (*N* = 35) and 4.2% (*N* = 76), respectively.

### Age and frailty of the cohort

The majority of patients scored 0 on the mFI (65.3%), with 25.9%, 8.5%, and 0.3% scoring 1, 2 and 3 points, respectively. The hypertension and diabetes mellitus components were the main contributors to the mFI scores, being present in 26.1% and 13.3% of patients, respectively. Only 0.3% (*N* = 5) of patients were non-independent with their activities of daily living. A significant correlation between age and mFI was observed (rho: 0.304, *p* < 0.001), with the median age increasing from 62.1 (IQR: 52.2–70.9) to 71.7 (64.4–76.7) years, for those with an mFI of 0 vs. 2–3. This was largely as a result of the comorbidity components of the mFI, with rates of hypertension, diabetes mellitus and COPD/pneumonia all increasing significantly with age (all *p* < 0.001).

### Characteristics and outcomes by age and frailty

For the initial analyses, both age and mFI were dichotomised, with age ≥ 75 years classified as “elderly” (18.6%; *N* = 340), and mFI ≥ 1 classified as “frail” (34.7%; *N* = 634). Of the demographic factors considered (Table 1), both elderly and frail patients were found to be significantly more likely to be male, and to have malignant tumours. Elderly patients were additionally found to have significantly less extensive resections, compared to the non-elderly (*p* = 0.042; minor resections: 57.6% vs. 52.1%), with a corresponding reduction in the average operative duration (median: 4.2 vs. 4.6 h; *p* < 0.001); no such significant differences were observed for the analysis of frailty (*p* = 0.200, *p* = 0.451, respectively). Rates of open (vs. laparoscopic) surgery were not found to differ significantly by age (*p* = 0.260) or frailty (*p* = 0.229). Univariable analysis of post-operative outcomes found overall complication rates to be significantly higher in both elderly (34.1% vs. 28.6%, *p* = 0.048) and frail (36.3% vs. 26.1%, *p* < 0.001) patients (Table 2). Further assessment of complication types found that this was largely a result of higher rates of medical complications (*p* < 0.001 for both), with surgical complication rates not found to be significantly higher in either elderly (*p* = 0.270) or frail (*p* = 0.061) patients. Elderly patients also had significantly longer lengths of stay (median: 7 vs. 6 days, *p* < 0.001), with no such difference observed for frail patients (median: 6 vs. 6 days, *p* = 0.060). Mortality rates were also significantly higher in the elderly and the frail, with 90-day mortality of 6.8% vs. 3.6% (*p* = 0.015) for elderly vs. non-elderly, and 6.6% vs. 2.9% (*p* < 0.001) for frail vs. non-frail.

**Table 1:** Cohort characteristics by age and frailty

	*N*	Age at surgery			mFI Score		
		< 75 years	≥ 75 years	*p*-value	mFI = 0	mFI ≥ 1	*p*-value
Age at surgery (years)	1826	62.2 (53.4–68.7)	78.6 (76.6–80.8)	N/A	62.1 (52.2–70.9)	69.7 (63.2–75.6)	**< 0.001**
Gender (% male)	1826	812 (54.6%)	222 (65.3%)	** < 0.001**	615 (51.6%)	419 (66.1%)	**< 0.001**
BMI (kg/m^2^)	1825	27.1 (24.3–30.9)	27.2 (24.7–30.0)	0.840	26.6 (23.9–29.8)	28.4 (25.5–31.8)	**< 0.001**
ASA grade (% > 2)	1826	421 (28.3%)	103 (30.3%)	0.466	345 (28.9%)	179 (28.2%)	0.786
Hypertension	1826	333 (22.4%)	144 (42.4%)	**< 0.001**	0 (0.0%)	477 (75.2%)	N/A
Diabetes mellitus	1826	177 (11.9%)	65 (19.1%)	**< 0.001**	0 (0.0%)	242 (38.2%)	N/A
Congestive heart failure	1826	2 (0.1%)	0 (0.0%)	1.000	0 (0.0%)	2 (0.3%)	N/A
COPD/pneumonia	1826	52 (3.5%)	22 (6.5%)	**0.021**	0 (0.0%)	74 (11.7%)	N/A
Non-independent in ADL	1826	4 (0.3%)	1 (0.3%)	1.000	0 (0.0%)	5 (0.8%)	N/A
mFI (% ≥ 1)	1826	456 (30.7%)	178 (52.4%)	** < 0.001**	–	–	–
Indication for surgery	1826			**< 0.001**			**< 0.001**
Colorectal liver metastases		848 (57.1%)	226 (66.5%)		705 (59.1%)	369 (58.2%)	
Hepatocellular carcinoma		115 (7.7%)	49 (14.4%)		75 (6.3%)	89 (14.0%)	
Gallbladder cancer/cholangiocarcinoma		149 (10.0%)	31 (9.1%)		102 (8.6%)	78 (12.3%)	
Other malignant		145 (9.8%)	18 (5.3%)		123 (10.3%)	40 (6.3%)	
Benign		229 (15.4%)	16 (4.7%)		187 (15.7%)	58 (9.1%)	
Extent of resection	1826			0.042**			0.200**
Minor		774 (52.1%)	196 (57.6%)		623 (52.3%)	347 (54.7%)	
Major		501 (33.7%)	107 (31.5%)		397 (33.3%)	211 (33.3%)	
Extra-major		211 (14.2%)	37 (10.9%)		172 (14.4%)	76 (12.0%)	
Operative approach (% open)	1826	1285 (86.5%)	286 (84.1%)	0.260	1034 (86.7%)	537 (84.7%)	0.229
Operative duration (hours)	1790	4.6 (3.6–5.8)	4.2 (3.4–5.2)	**< 0.001**	4.5 (3.5–5.7)	4.4 (3.5–5.6)	0.451
Blood transfusion*	1826	147 (9.9%)	43 (12.6%)	0.140	119 (10.0%)	71 (11.2%)	0.422

Continuous variables are reported as median (interquartile range), with *p*-values from Mann–Whitney U tests. Categorical variables are reported as *N* (column %), with *p*-values from Fisher’s exact tests, unless stated otherwise. Bold *p*-values are significant at *p* < 0.05. *Within 24 h post-operatively. **p-Value from Mann–Whitney U test, as the factor is ordinal. *ADL* activities of daily living; *BMI* body mass index; *COPD* Chronic obstructive pulmonary disease; *mFI* modified frailty index

**Table 2:** Post-operative complications by patient age and modified Frailty Index

	Age at surgery			mFI Score		
	< 75 years	≥ 75 years	*p*-value	mFI = 0	mFI ≥ 1	*p*-value
Any complications	425 (28.6%)	116 (34.1%)	**0.048**	311 (26.1%)	230 (36.3%)	** < 0.001**
Highest Clavien–Dindo grade			0.032**			< 0.001**
No complication	1061 (71.4%)	224 (65.9%)		881 (73.9%)	404 (63.7%)	
Grade I–II	270 (18.2%)	75 (22.1%)		201 (16.9%)	144 (22.7%)	
Grade III–V	155 (10.4%)	41 (12.1%)		110 (9.2%)	86 (13.6%)	
Length of stay (days)*	6 (5–8)	7 (5–9)	**< 0.001**	6 (5–8)	6 (5–9)	0.060
30-day mortality	22 (1.5%)	13 (3.8%)	**0.008**	14 (1.2%)	21 (3.3%)	**0.002**
90-day mortality	53 (3.6%)	23 (6.8%)	**0.015**	34 (2.9%)	42 (6.6%)	**< 0.001**
In-hospital mortality	25 (1.7%)	15 (4.4%)	**0.006**	17 (1.4%)	23 (3.6%)	**0.004**
Details of surgical complications						
PHLF	45 (3.0%)	7 (2.1%)	0.468	28 (2.3%)	24 (3.8%)	0.103
PHLF Grade			0.335**			0.079**
No PHLF	1441 (97.0%)	333 (97.9%)		1164 (97.7%)	610 (96.2%)	
Grade A	24 (1.6%)	3 (0.9%)		14 (1.2%)	13 (2.1%)	
Grade B	8 (0.5%)	2 (0.6%)		7 (0.6%)	3 (0.5%)	
Grade C	13 (0.9%)	2 (0.6%)		7 (0.6%)	8 (1.3%)	
Bile leak	65 (4.4%)	9 (2.6%)	0.170	41 (3.4%)	33 (5.2%)	0.081
Post-operative haemorrhage	25 (1.7%)	8 (2.4%)	0.372	23 (1.9%)	10 (1.6%)	0.713
Wound infection	45 (3.0%)	10 (2.9%)	1.000	37 (3.1%)	18 (2.8%)	0.886
Re-operation for bleeding	10 (0.7%)	5 (1.5%)	0.175	11 (0.9%)	4 (0.6%)	0.597
Radiological drainage of collection	42 (2.8%)	6 (1.8%)	0.348	28 (2.3%)	20 (3.2%)	0.357
Other surgical complication	32 (2.2%)	5 (1.5%)	0.526	20 (1.7%)	17 (2.7%)	0.164
Any of the above	226 (15.2%)	43 (12.6%)	0.270	162 (13.6%)	107 (16.9%)	0.061
Details of medical complications						
Pneumonia	85 (5.7%)	20 (5.9%)	0.897	62 (5.2%)	43 (6.8%)	0.171
AKI (requiring RRT)	4 (0.3%)	2 (0.6%)	0.311	3 (0.3%)	3 (0.5%)	0.424
Respiratory failure (requiring vent.)	12 (0.8%)	5 (1.5%)	0.342	10 (0.8%)	7 (1.1%)	0.613
Myocardial infarction	5 (0.3%)	3 (0.9%)	0.173	6 (0.5%)	2 (0.3%)	0.722
Dysrhythmia	35 (2.4%)	20 (5.9%)	**0.001**	30 (2.5%)	25 (3.9%)	0.113
Other medical complication	120 (8.1%)	40 (11.8%)	**0.034**	84 (7.0%)	76 (12.0%)	**< 0.001**
Any of the above	247 (16.6%)	84 (24.7%)	**< 0.001**	182 (15.3%)	149 (23.5%)	**< 0.001**

Continuous variables are reported as median (interquartile range), with *p*-values from Mann–Whitney U tests. Categorical variables are reported as *N* (column %), with *p*-values from Fisher’s exact tests, unless stated otherwise. Bold *p*-values are significant at *p* < 0.05. *Excludes patients that died in hospital prior to discharge. **p-Value from Mann–Whitney U test, as the factor is ordinal. *AKI* acute kidney injury; *mFI* modified frailty index; *PHLF* Post hepatectomy liver failure; *RRT* renal replacement therapy; *Vent*. ventilation

### Interplay between age and frailty

Comparisons of outcomes between frail and non-frail patients were then performed within the elderly and non-elderly patient subgroups. Analysis of mortality, complication rates and length of stay found the effect of frailty to be similar in non-elderly and elderly patients (Table 3). For example, for non-elderly patients, frailty (vs. non-frailty) was associated with odds ratio for 90-day mortality of 2.25 (5.7% vs. 2.6%), which was similar to the 2.19 (9.0% vs. 4.3%) observed in elderly patients (*p* = 0.961).

**Table 3:** Interplay between age and frailty

	Age: < 75 years			Age: ≥ 75 years			Interaction term *p*-value
	mFI = 0 (*N* = 1030)	mFI ≥ 1 (*N* = 456)	Odds ratio (95% CI)	mFI = 0 (*N* = 162)	mFI ≥ 1 (*N* = 178)	Odds ratio (95% CI)	
Any complications	265 (25.7%)	160 (35.1%)	1.56 (1.23–1.98)	46 (28.4%)	70 (39.3%)	1.63 (1.04–2.58)	0.860
Any surgical complication	143 (13.9%)	83 (18.2%)	1.38 (1.03–1.86)	19 (11.7%)	24 (13.5%)	1.17 (0.62–2.23)	0.652
Any medical complication	151 (14.7%)	96 (21.1%)	1.55 (1.17–2.06)	31 (19.1%)	53 (29.8%)	1.79 (1.08–2.97)	0.628
Length of stay (days)*	6.3 (6.1, 6.5)*	6.5 (6.2, 6.9)*	4% (− 3%, 10%)	6.9 (6.3, 7.6)*	7.8 (7.0, 8.6)*	12% (− 2%, 28%)*	0.276*
In-hospital mortality	13 (1.3%)	12 (2.6%)	2.11 (0.96–4.67)	4 (2.5%)	11 (6.2%)	2.60 (0.81–8.34)	0.773
30-day mortality	10 (1.0%)	12 (2.6%)	2.76 (1.18–6.43)	4 (2.5%)	9 (5.1%)	2.10 (0.64–6.97)	0.718
90-day mortality	27 (2.6%)	26 (5.7%)	2.25 (1.30–3.89)	7 (4.3%)	16 (9.0%)	2.19 (0.88–5.46)	0.961

For binary outcomes, odds ratios are from binary logistic regression models within each age subgroup, with mFI (≥ 1 vs. 0) as the independent variable. The *p*-values relate to the interaction terms of binary logistic regression models with age, frailty and the age*frailty interaction as independent variables, hence represent a comparison between the reported odds ratio for each subgroup. *Analysis of length of stay excluded those patients who died in hospital prior to discharge. For the remainder, the average lengths of stay are reported as geometric mean (95% CI). Lengths of stay were then log_10_-transformed, and analysed using an ANOVA model, parameterised as previously described, with the comparisons between groups reported as percentage differences. *CI* confidence interval; *mFI* modified frailty index

### Associations with primary outcomes

**Fig. 1:**
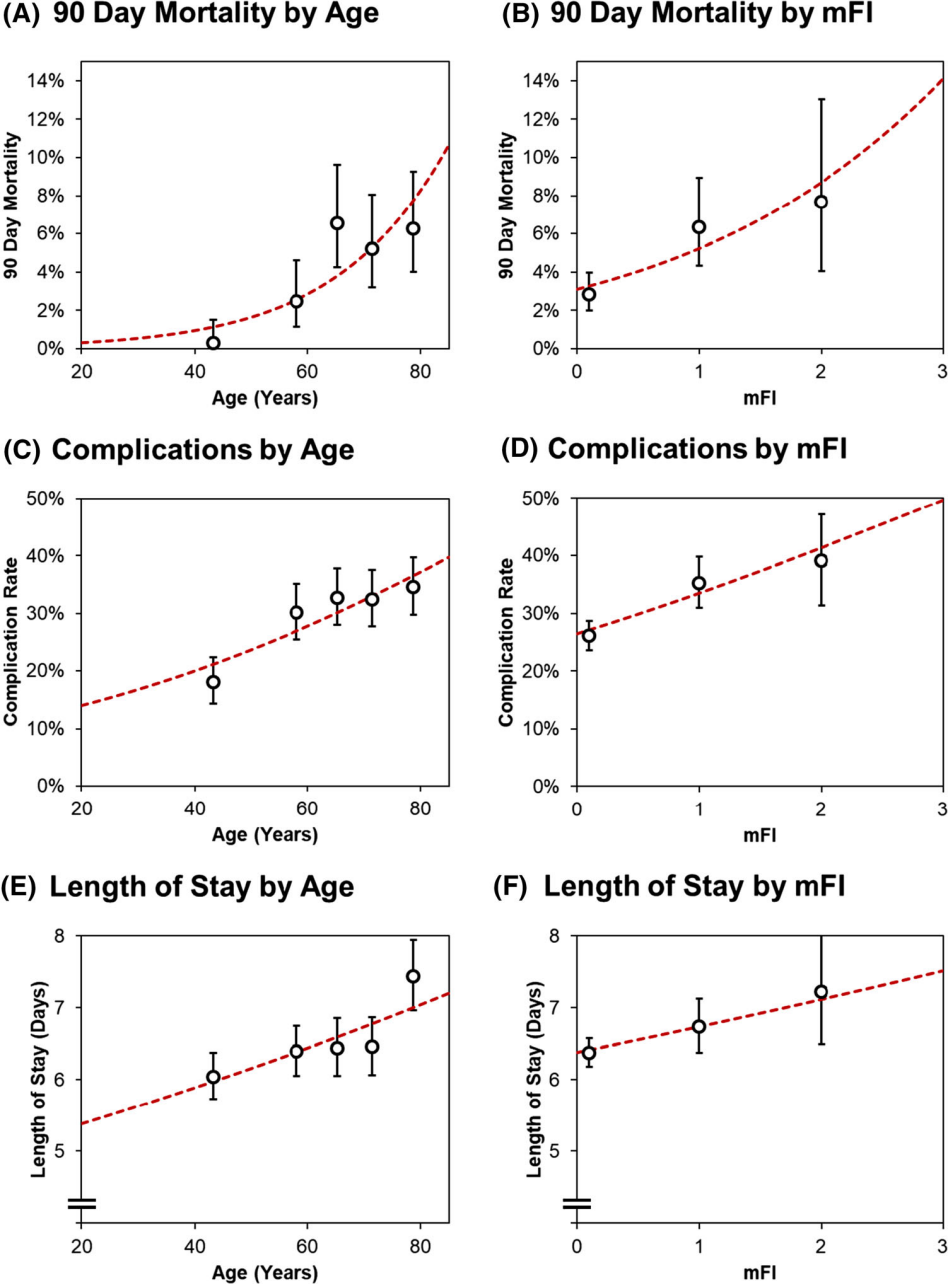
Associations between age/mFI and the primary outcomes. Points represent the observed mortality/complication rates, or the geometric mean lengths of stay, with whiskers representing 95% confidence intervals. For age, each point corresponds to a quintile of the age distribution, and is plotted at the mean of the interval, whilst points for mFI correspond to values of 0, 1 and 2; mFI = 3 was not plotted, due to the small sample size (*N* = 5). Trend lines are from univariable binary logistic regression models for the dichotomous outcomes, with a univariable log-linear regression model used for length of stay, as per Table 4

The associations between both age and frailty, and the primary outcomes of 90-day mortality, post-operative complications and length of stay were then assessed in further detail. Age and mFI were treated as continuous covariates in these analyses and were found to be significantly associated with all three outcomes on univariable analysis (Fig. 1). On multivariable analysis, both age and mFI were found to be significant independent predictors of 90-day mortality, with odds ratios of 1.71 (95% CI: 1.32–2.22, *p* < 0.001) per decade of age and 1.45 (1.04–2.03, *p* = 0.029) per point on the mFI (Table 4). Age and mFI were found to be significant independent predictors of post-operative complications (*p* < 0.001, *p* = 0.002, respectively). Whilst age was found to be significantly associated with length of stay (*p* < 0.001), mFI narrowly missed statistical significance in this analysis (*p* = 0.056).

**Table 4:** Associations between age/frailty and primary outcomes

	Univariable analysis		Multivariable analysis	
	Coefficient (95% CI)	*p*-value	Coefficient (95% CI)	*p*-value
*90-day mortality*				
Age at surgery (per decade)	1.75 (1.39–2.22)	**< 0.001**	1.71 (1.32–2.22)	**< 0.001**
mFI (per point)	1.72 (1.28–2.32)	**< 0.001**	1.45 (1.04–2.03)	**0.029**
Any post-operative complications				
Age at surgery (per decade)	1.24 (1.14–1.35)	**< 0.001**	1.26 (1.14–1.39)	**< 0.001**
mFI (per point)	1.40 (1.21–1.62)	**< 0.001**	1.30 (1.10–1.53)	**0.002**
*Length of stay*				
Age at surgery (per decade)	4.5% (2.4%, 6.7%)	**< 0.001**	5.9% (3.7%, 8.1%)	**< 0.001**
mFI (per point)	5.5% (1.4%, 9.9%)	**0.009**	3.8% (− 0.1%, 8.0%)	0.056

Analyses of 90-day mortality and complications were performed using binary logistic regression models, with coefficients representing odds ratios. Analysis of length of stay was performed using general linear models, with the log_10_[length of stay] as the dependent variable; coefficients represent percentage differences. For all outcomes, age and mFI were initially treated as continuous covariates in separate univariable models, with coefficients representing the change in the outcome per decade or per point, respectively. Multivariable models were then produced, which considered gender, BMI, ASA grade, indication for surgery, extent of resection, operative approach and duration of surgery for inclusion, with a backwards stepwise approach to variable selection. The full models are reported in Supplementary Table 1–3. Bold *p*-values are significant at *p* < 0.05. *CI* confidence interval; *mFI* modified frailty index; *OR* odds ratio

### Effect of operative approach by age and frailty in minor resections

The operative approach was found to vary significantly by the extent of the resection (*p* < 0.001), with 22.3% of minor resections being laparoscopic, compared to 6.1% of major and 0.8% of extra-major resections. As such, to negate the confounding effect of the extent of resection, only the subgroup of minor resections were considered in the analysis (*N* = 970). Within this subgroup, neither complication nor mortality rates were found to differ significantly by the operative approach (Table 5). However, open surgery was associated with a significantly longer operative duration (median: 4.1 vs. 3.6 h, *p* < 0.001) and length of stay (median 6 vs. 4 days, *p* < 0.001), compared to laparoscopic surgery. The effects of operative approach were then compared between elderly vs. non-elderly and frail vs. non-frail patients (Table 6). We found no evidence to suggest that the effect of operative approach varied significantly by age or frailty for any of the outcomes considered. For example, open (vs. laparoscopic) surgery was associated with a 53% (geometric mean: 6.5 vs. 4.2 days) longer length of stay in frail patients, which was similar to the 58% (6.1 vs. 3.8 days) longer length of stay after open (vs. laparoscopic) surgery in non-frail patients (*p* = 0.699). As such, the relative benefits of laparoscopic vs. open surgery appeared to be similar, regardless of the age or frailty of the patient. However, these analyses were limited by the small number of outcomes occurring within some subgroups of patients, with no events being observed for some of the outcomes considered. This will have resulted in very low statistical power, meaning that only large differences will have been detectable. As such, this analysis will be subject to an inflated false-negative rate, and the results must be interpreted with this in mind.

**Table 5:** Outcomes by operative approach in minor resections

	Operative approach		
	Laparoscopic (*N* = 216)	Open (*N* = 754)	*p*-value
Operative duration (hours)	3.6 (3.0–4.4)	4.1 (3.3–5.2)	**< 0.001**
Any complications	37 (17.1%)	175 (23.2%)	0.062
Any surgical complication	13 (6.0%)	74 (9.8%)	0.104
Any medical complication	24 (11.1%)	110 (14.6%)	0.219
Length of stay (days)*	4 (3–5)	6 (5–7)	**< 0.001**
In-hospital mortality	2 (0.9%)	7 (0.9%)	1.000
30-day mortality	2 (0.9%)	6 (0.8%)	1.000
90-day mortality	3 (1.4%)	19 (2.5%)	0.441

Only those patients with minor resections (*N* = 970) are included in the analysis. Data are reported as *N* (column %), with *p*-values from Fisher’s exact tests, or as median (interquartile range), with *p*-values from Mann–Whitney U tests. Bold *p*-values are significant at *p* < 0.05. *Analysis of length of stay excluded those patients who died in hospital prior to discharge

**Table 6:** Interplay between operative approach and age/frailty in minor resections

	Age: < 75 years			Age: ≥ 75 years			
	Laparoscopic (*N* = 172)	Open (*N* = 602)	Odds ratio (95% CI)	Laparoscopic (*N* = 44)	Open (*N* = 152)	Odds ratio (95% CI)	Interaction term *p*-value
Operative duration (hours)*	3.7 (3.5, 3.9)*	4.2 (4.1, 4.4)*	15% (8%, 22%)*	3.8 (3.5, 4.2)*	3.9 (3.8, 4.1)*	3% (− 6%, 14%)*	0.096*
Any complications	29 (16.9%)	132 (21.9%)	1.38 (0.89–2.16)	8 (18.2%)	43 (28.3%)	1.78 (0.76–4.13)	0.610
Any surgical complication	11 (6.4%)	60 (10.0%)	1.62 (0.83–3.16)	2 (4.5%)	14 (9.2%)	2.13 (0.47–9.75)	0.747
Any medical complication	18 (10.5%)	79 (13.1%)	1.29 (0.75–2.22)	6 (13.6%)	31 (20.4%)	1.62 (0.63–4.18)	0.683
Length of stay (days)*	3.9 (3.6, 4.2)*	6.0 (5.8, 6.3)*	54% (41%, 67%)*	4.4 (3.7, 5.1)*	7.1 (6.5, 7.7)*	61% (34%, 92%)*	0.632*
In-hospital mortality	0 (0.0%)	4 (0.7%)	NC**	2 (4.5%)	3 (2.0%)	0.42 (0.07–2.61)	NC**
30-day mortality	0 (0.0%)	3 (0.5%)	NC**	2 (4.5%)	3 (2.0%)	0.42 (0.07–2.61)	NC**
90-day mortality	1 (0.6%)	15 (2.5%)	4.37 (0.57–33.3)	2 (4.5%)	4 (2.6%)	0.57 (0.10–3.21)	0.134

	mFI = 0				mFI ≥ 1		
	Laparoscopic (*N* = 130)	Open (*N* = 493)	Odds ratio (95% CI)	Laparoscopic (*N* = 86)	Open (*N* = 261)	Odds ratio (95% CI)	Interaction term *p*-value
Operative duration (hours)*	3.6 (3.4, 3.8)*	4.2 (4.1, 4.3)*	17% (10%, 25%)*	3.9 (3.6, 4.2)*	4.1 (4.0, 4.3)*	6% (− 2%, 15%)*	0.066*
Any complications	21 (16.2%)	97 (19.7%)	1.27 (0.76–2.13)	16 (18.6%)	78 (29.9%)	1.86 (1.02–3.41)	0.345
Any surgical complication	8 (6.2%)	42 (8.5%)	1.42 (0.65–3.10)	5 (5.8%)	32 (12.3%)	2.26 (0.85–6.01)	0.465
Any medical complication	13 (10.0%)	61 (12.4%)	1.27 (0.68–2.39)	11 (12.8%)	49 (18.8%)	1.58 (0.78–3.19)	0.656
Length of stay (days)*	3.8 (3.5, 4.2)*	6.1 (5.8, 6.3)*	58% (43%, 73%)*	4.2 (3.8, 4.8)*	6.5 (6.1, 6.9)*	53% (34%, 74%)*	0.699*
In-hospital mortality	1 (0.8%)	3 (0.6%)	0.79 (0.08–7.66)	1 (1.2%)	4 (1.5%)	1.32 (0.15–12.00)	0.749
30-day mortality	1 (0.8%)	3 (0.6%)	0.79 (0.08–7.66)	1 (1.2%)	3 (1.1%)	0.99 (0.10–9.63)	0.891
90-day mortality	1 (0.8%)	9 (1.8%)	2.40 (0.30–19.1)	2 (2.3%)	10 (3.8%)	1.67 (0.36–7.79)	0.785

Only those patients with minor resections (*N* = 970) are included in the analysis, with analysis of length of stay additionally excluding those patients who died in hospital prior to discharge. For the analysis by age, odds ratios are from binary logistic regression models within each age subgroup, with the operative approach (open vs. laparoscopic) as the independent variable. The *p*-values relate to the interaction terms of binary logistic regression models with age, operative approach and the age*operative approach interaction as independent variables, hence represent a comparison between the reported odds ratio for each subgroup. The analysis was repeated similarly for mFI. *Operative duration and length of stay followed skewed distributions, hence were log_10_-transformed, and analysed using an ANOVA model, parameterised as previously described; averages are reported as geometric means (95% CI), and comparisons between groups are reported as percentage differences. **Hazard ratios were not calculable, as there were no events in one of the subgroups

## Discussion

Frailty is increasingly recognised as being an important determinant of post-operative outcomes in surgical patients [18], and our data have shown that frailty is an independent predictor of morbidity and mortality after hepatectomy. Over one third of patients in this large cohort were considered frail, and despite acceptable short-term outcomes, were exposed to an increased risk of medical complications, prolonged recovery and post-operative mortality compared to non-frail patients, which is consistent with published data [9, 19, 20]. Importantly, frailty was associated with worse outcomes for both elderly and non-elderly patients undergoing hepatectomy. Data from meta-analyses and randomized trials have shown that laparoscopic hepatectomy is associated with improved short-term outcomes compared to open hepatectomy [2, 21]. Our analysis has indicated that the short-term benefits (e.g. reduced hospital stay) of laparoscopic surgery are retained in frail patients undergoing minor hepatectomy, and suggests that in carefully selected patients, frailty is not a contraindication to laparoscopic minor hepatectomy. This finding is not unexpected, since the benefits of laparoscopic surgery are primarily due to reduced medical complications, which occur as a result of less post-operative pain, improved respiratory function and earlier ambulation. It is unknown whether frail patients undergoing major hepatectomy would also benefit from a laparoscopic approach. A recent patient blinded randomized trial demonstrated a faster recovery in patients undergoing laparoscopic major hepatectomy compared to open [22], but there is currently no data specifically evaluating the outcomes of laparoscopic major hepatectomy in frail patients. Laparoscopic major hepatectomy is a complex procedure associated with significantly longer operating times and longer hepatic inflow occlusion times compared to open surgery, and it is unknown whether these factors may negate the potential benefits of laparoscopic surgery in frail patients. It was not possible to evaluate the effect of laparoscopic approach in frail patients undergoing major hepatectomy in our study due to small numbers in this subgroup (Supplementary Table 4), and it is likely that a multi-centre study would be required.

Detection of frailty prior to major surgery is important, since it may allow risk stratification, facilitates preoperative counselling, and guides perioperative management including choice of post-operative destination (i.e. critical care or surgical high dependency). Frail patients may also benefit from preoperative interventions to address reversible deficits (e.g. aerobic fitness and nutrition) [23], and the concept of prehabilitation is likely to become a central component of perioperative care for patients being considered for hepatectomy in the near future [24, 25]. Post-operative functional recovery following hospital discharge and return to baseline function is an under-researched, patient-centred outcome that may also be influenced by frailty. A recent Japanese study of over 65-year-old patients undergoing hepatectomy found that frailty, advanced age (≥ 76 years) and open hepatectomy were independent risk factors for post-operative loss of independence [9].

This study has several limitations. Due to its retrospective nature, the effect of selection bias on the operative approach may have affected the results, and it was also not possible to ascertain how many patients were deemed unsuitable for hepatectomy due to severe frailty. As discussed above, the small number of laparoscopic major hepatectomy patients precluded analysis of the impact of frailty and outcome in this subgroup.

In conclusion, frailty is a common finding in patients undergoing hepatectomy, and is an independent risk factors for post-operative morbidity and mortality. The short-term benefits of laparoscopic hepatectomy appear to be preserved in frail patients. As such, frailty is not contraindicated in patients being considered for laparoscopic minor hepatectomy. Further study is needed to determine if frail patients would also benefit from laparoscopic major hepatectomy.
